# Exosomal hsa_circ_0004658 derived from RBPJ overexpressed-macrophages inhibits hepatocellular carcinoma progression via miR-499b-5p/JAM3

**DOI:** 10.1038/s41419-021-04345-9

**Published:** 2022-01-10

**Authors:** Lei Zhang, Jing Zhang, Pengfei Li, Ting Li, Zhiqin Zhou, Huiling Wu

**Affiliations:** 1 Department of Plastic and Aesthetic Center, the First Affiliated Hospital, College of Medicine, Zhejiang University, Hangzhou, Zhejiang China; 2 College of Medicine, Zhejiang University, Hangzhou, Zhejiang China

**Keywords:** Cancer stem cells, Liver cancer

## Abstract

Macrophage-derived exosomes (Mφ-Exo) have multidimensional involvement in tumor initiation, progression, and metastasis, but their regulation in hepatocellular carcinoma (HCC) is not fully understood. RBPJ has been implicated in macrophage activation and plasticity. In this study we assess the role of exosomes derived from RBPJ-overexpressed macrophages (RBPJ^+/+^ Mφ-Exo) in HCC. The circular RNA (circRNA) profiles in RBPJ^+/+^ Mφ-Exo and THP-1-like macrophages (WT Mφ)-Exo was evaluated using circRNA microarray. CCK-8, Transwell, and flow cytometry analyses were used to evaluate the function of Mφ-Exo-circRNA on HCC cells. Luciferase reporter assays, RNA immunoprecipitation, and Pearson’s correlation analysis were used to confirm interactions. A nude mouse xenograft model was used to further analyze the functional significance of Mφ-Exo-cirRNA in vivo. Our results shown that hsa_circ_0004658 is upregulated in RBPJ^+/+^ Mφ-Exo compared to WT Mφ-Exo. RBPJ^+/+^ Mφ-Exo and hsa_circ_0004658 inhibits proliferation and promotes apoptosis in HCC cells, whereas hsa_circ_0004658 knockdown stimulated cell proliferation and migration but restrained apoptosis in vitro and promotes tumor growth in vivo. The effects of RBPJ^+/+^ Mφ-Exo on HCC cells can be reversed by the hsa_circ_0004658 knockdown. Mechanistic investigations revealed that hsa_circ_0004658 acts as a ceRNA of miR-499b-5p, resulting in the de-repression of JAM3. These results indicate that exosome circRNAs secreted from RBPJ^+/+^ Mφ inhibits tumor progression through the hsa_circ_0004658/miR-499b-5p/JAM3 pathway and hsa_circ_0004658 may be a diagnostic biomarker and potential target for HCC therapy.

## Introduction

Hepatocellular carcinoma (HCC) has become the second leading cause of cancer mortality worldwide, predominately because of its association with chronic liver disease [1, 2]. Despite the evolution of targeted therapies, such as sorafenib, the treatment of HCC remains a challenge because of the heterogeneity of tumors and low tolerance to therapies caused by liver cirrhosis [3–5]. Tumor-associated macrophages are the most abundant cell-type in the HCC tumoral environment [6]. They regulate immune responses to promote cancer-related inflammation [7, 8], the stimulation of angiogenesis [9], and tissue remodeling [10], and may have potential in therapy and marker development [11].

Injury to the liver results in Kupffer cell activation, which leads to the release of inflammatory cytokines and chemokines, and the infiltration of monocytes [12]. The monocytes generate an abundance of inflammatory macrophages, which possess a great deal of plasticity and adapt their function to that demanded by signals in the immediate microenvironment [10]. The functions following macrophage induction can perpetuate hepatic injury and tumor development by activating fibrosis and angiogenesis [13, 14]. However, macrophages also possess the ability to inhibit fibrosis and promote tissue repair but the exact processes by which this is regulated is unclear. Increasing evidence implicates that exosomes mediate the interactions between macrophages and cancer cells [15–18]. Macrophage-derived exosomes were found to promote the proliferation and metastasis of breast cancer cells [15]. Exosomes from macrophages exposed to apoptotic breast cancer cells contain higher levels of IL-6, which is thought to increase the phosphorylation of STAT3 and the subsequent regulation of genes involved in proliferation. Moreover, macrophage-derived exosomes also contribute to the progression of pancreatic ductal adenocarcinoma through the microRNA (miRNA) regulation of the TGF-β signaling pathway [17] and, in ovarian cancer, exosomal miRNAs derived from macrophages promote drug resistance through the PTEN-PI3K/AKT pathway [18]. Circular RNA (circRNA) have also been found in exosomes [19]. CircRNA are thought to modulate the expression of genes and miRNA. Recent evidence suggests that circRNAs may be involved in the fine regulation of miRNA in HCC by competitive binding or sponging [20–23]. For instance, hsa_circ_0091570 was found to interact with miR-1307 in HCC and the inhibition of hsa_circ_0091570 promoted cell proliferation and reduced cell apoptosis [20]. However, the exact involvement of macrophage-derived exosomes, miRNAs, and circRNAs in the progression of HCC is unclear.

The Notch pathway is involved in several essential cellular processes, such as proliferation and development, and is also believed to be responsible for the activation and differentiation of macrophages [24, 25]. The recombination signal binding protein-Jκ (RBPJ) is a transcriptional regulator that is often used as a marker to indicate the activation of Notch signaling [26]. Notch intracellular domains are released by Notch ligands and translocate to the nucleus where they bind to RBPJ [27]. The inhibition of the Notch signaling pathway through the disruption of RBPJ has been found to alleviate hepatic fibrosis through preventing NF-κB activation [28]. Moreover, Notch-RBPJ signaling was found to regulate TLR-induced inflammatory macrophage polarization by the indirect regulation of M1-specific genes [29]. Furthermore, recent results suggest that Notch signaling can regulate different subsets of tumor-associated macrophages in HCC [30].

In this study, we investigate the effects of macrophage-derived exosomes overexpressing RBPJ (RBPJ^+/+^ Mφ-Exo) on proliferation and apoptosis in HCC cells and compared them to exosomes from THP-1-like macrophage (WT Mφ-Exo) [31]. To further understand the regulatory mechanism of RBPJ^+/+^ Mφ-Exo in HCC, we also determine the circRNA that are differentially regulated when RBPJ is upregulated in Mφ-Exo. In addition, we have identified the miRNA binding partners of the circRNA upregulated in RBPJ^+/+^ Mφ-Exo and their targets. The aim of this study was to identify pathways that are uniquely expressed in HCC progression, further understand the mechanisms of macrophage-derived exosomes-circRNA, and to identify diagnostic biomarkers and potential therapeutic targets.

## Materials and methods

### Cell culture and clinical specimens

Human monocytic cell line THP-1 and HCC cell lines (SMMC-7721and HepG2) were purchased from ATCC and maintained according to ATCC guidelines. THP-1 cells were cultured in RPMI-1640 medium (Gibco, Carlsbad, CA, USA), and HCC cells were cultured in Dulbecco’s Modified Eagle medium (DMEM, Gibco, USA) containing 10% heat-inactivated fetal bovine serum (FBS) (Thermo Fisher Scientific, Waltham, MA, USA), 100 U/mL penicillin, and 100 µg/mL streptomycin (HyClone Laboratories, Logan, UT, USA). Cells were cultured at 37 °C in a humidified incubator with 5% CO_2_ and were used in the exponential growth phase.

A total of 50 paired HCC tissues and para-carcinoma normal tissues were obtained from patients who underwent surgery at the First Affiliated Hospital, College of Medicine, Zhejiang University between 2013 and 2019. All the patients were diagnosed by histopathology and did not receive any other treatment prior to the operation. Written informed consent was obtained from all patients before the research. This study was approved by the Ethics Committees of the First Affiliated Hospital, College of Medicine at Zhejiang University and conducted in accordance with the Helsinki Declaration.

### Isolation of exosomes derived from THP-1 Mφ cells with or without the overexpression of RBPJ

To obtain WT Mφ and RBPJ^+/+^ Mφ, THP-1 cells were transfected with the pCMV6 empty vector or pCMV6 overexpressing RBPJ (OriGene, Rockville, MD, USA) and seeded at a density of 0.5 × 10^6^ cells per well in a six-well culture plate. Cells were stimulated with 200 ng/mL of phorbol 12-myristate 13-acetate (PMA, Sigma-Aldrich, St Louis, MO, USA) for 24 h. Gradient centrifugation was used to isolate exosomes from the medium of the cell cultures. The medium was first centrifuged at 3000 × *g* for 30 min to remove cells and cellular debris. The supernatant was collected and centrifuged again at 10,000 × *g* for 30 min to remove larger microvesicles. Finally, exosomes were isolated from the supernatant at 110,000 × *g* for 70 min at 4 °C and stored in PBS at −80 °C.

### Transmission electron microscopy assay

Exosomes for transmission electron microscopy (TEM) were prepared as described previously [32]. Briefly, exosomes were first fixed in 2.5% glutaraldehyde pH 7.2 at 4 °C. They were then washed in PBS and fixed in 1% osmium tetroxide for 60 min at room temperature before being embedded in 10% gelatin. The embedded exosomes were cut into 1 mm blocks and dehydrated with increasing concentrations of alcohol. The alcohol was then exchanged by increasing concentrations of Quetol-812 epoxy resin mixed with propylene oxide. Samples were finally embedded in Quetol-812 epoxy resin, polymerized at a temperature gradient, and cut into ultrathin sections using a Leica UC6 ultramicrotome. Sections were stained with uranyl acetate and lead citrate for observations under a transmission electron microscope.

### Microarray analysis

Total RNA was isolated using Trizol reagent (Life Technologies, Camarillo, CA, USA) and was quantified using a NanoDrop ND-1000. To enrich circRNA and remove linear RNA, the RNA was digested with Rnase R (Epicentre, Madison, WI, USA). The enriched circRNA was then amplified and labeled fluorescently using a Super RNA Labeling kit (Arraystar, Rockville, MD, USA) following the manufacturer’s instructions. The labeled cRNA was hybridized to an Arraystar Human circRNA Array V2 (8 × 15 K). Slides were then washed and scanned with an Agilent Scanner G2505C. Acquired images were analyzed with Agilent Feature Extraction software (version 11.0.1.1). The limma package in R was used for quantile normalization and to process the data. Fold change filtering and hierarchical clustering was used to identify differentially expressed circRNA and expression patterns.

### Quantitative RT-PCR analysis (qRT-PCR)

To perform qRT-PCR, a Prime Script RT reagent kit (Takara, Beijing, China) was used to synthesize cDNA from the total RNA and SYBR Premix Ex Taq was used to perform RT-PCR in a CFX96 Real-Time PCR system (Bio-Rad, Hercules, CA, USA). Glyceraldehyde-3-phosphate dehydrogenase (GAPDH) was used as the intrinsic control for circRNA and mRNA, and U6 was used as the endogenous control for miRNA. The primer sequences were as follows: hsa_circ_0004658: Forward primer (5′-GCAAGATCTGGTCAAATTTCAG-3′), Reverse primer (5′- GCTCTGAGAATTGGCTGGG-3′); miR-499b-5p: Forward primer (5′-AGCAGAAAGTACACATAAACACA-3′), Reverse primer (5′-AGAGTTCTGCCACTATGTTTCA-3′); JAM3: Forward primer (5′-TCCAGCAATCGAACCCCAG-3′), Reverse primer (5′-CTTGTCTGCGAATCCGTAATGAT-3′); U6 mRNA: Forward primer (5′-CTCGCTTCGGCAGCACA-3′), Reverse primer (5′- ACGCTTCACGAATTTGC-3′); GAPDH mRNA: Forward primer (5′- AGAAGGCTGGGGCTCATTTG-3′), Reverse primer (5′-AGGGGCCATCCACAGTCTTC-3′). The relative change in expression was determined using the 2^−ΔΔCt^ method.

### Cell transfection

CircRNA (hsa_circ_0004658) overexpressed plasmid (p-circRNA) and the mock plasmid pcDNA3.1, small interfering RNAs (siRNAs) targeting circRNA and non-specific negative control oligos (si-control), miR-499b-5p mimic, inhibitor and the negative control (NC) were purchased from GenePharma (Shanghai, China). The lentivirus targeting hsa_circ_0004658 was purchased from GeneChem (Shanghai, China). SMMC-7721 and HepG2 cell lines were seeded in 6-well plates 24 h prior to pcDNA3.1, p-circRNA, si-control, si-circRNA, miR-499b-5p mimic or inhibitor transfection with 50–60% confluence, and then were transfected with Lipofectamine 3000 (Invitrogen) following the manufacturer’s instructions. The effects of knockdown or overexpression were examined by RT-qPCR using RNA extracted 48 h after transfection. For exosome treatment, HCC cells were cultured in medium containing 5 μg/ml exosome from WT Mφ, RBPJ^+/+^ Mφ or si-circRNA and RBPJ^+/+^ Mφ

### Cell proliferation, apoptotic, and migration assays

To assess cell proliferation, 2 × 10^3^ cells were seeded into each well of a 96-well plate. A cell counting kit (CCK)−8 (Beyotime Biotechnology, Nantong, China) was used to assess cell numbers according to manufacturer’s instructions. To measure apoptosis, cells were incubated in 24-well plates for 48 h and collected in flow tubes. Cells were then washed in PBS and 1× binding buffer three times, and stained using an Annexin V-FITC Apoptosis Detection Kit (Vazyme, Nanjing, China). The percentage of apoptosis was analyzed by flow cytometry (FACScan, BD Biosciences, USA). Migration was assessed using a Transwell system. First, cells were seeded into the upper compartment of the chamber (Millipore, Burlington, MA, USA) and the full culture medium was added to the lower chamber. After 24 h, the cells on the lower surface of the chamber were fixed with methanol and stained with 0.1% crystal violet. Five visual fields (200×) were randomly selected and the percentage of migrating cells was determined under microscope. All of the experiments were performed in triplicate.

### Luciferase reporter assay

Sequences of WT or mutant hsa_circ_0004658 or the full length of the 3′-UTR of JAM3 containing WT or mutated putative binding sites were inserted into the pmir-GLO vector (Promega Corp., Madison, WI, USA). to validate interactions. HCC cells were first seeded on 96-well plates and co-transfected with luciferase vectors (100 ng) and miR-499b-5p mimics/NC (200 pmol) using Lipofectamine 2000 transfection reagent (Invitrogen) following manufacturer’s instructions. Levels of luciferase activity were detected 48 h after transfection using a Dual-Luciferase reporter assay system (Promega, Madison, WI, USA) according to the manufacturer’s instructions. All the experiments were performed in triplicate.

### RNA immunoprecipitation (RIP) assay

We carried out the RIP assay using a Magna RIP RNA Binding Protein immunoprecipitation Kit (Millipore, Bedford, MA, USA) according to the manufacturer’s instructions. Briefly, cells (2 × 10^7^) were lysed with the lysis buffer provided in the kit and the lysate was divided between two tubes one containing anti-Argonaute2 (AGO2) antibody and the other a non-specific anti-IgG antibody (Millipore). The cell lysates were incubated overnight at 4 °C and then magnetic beads were added and the incubation continued for a further hour. Proteinase K was then added and samples were incubated at 55 °C for another hour. RNA extraction reagent (Solarbio, Beijing, China) was used to obtain the RNA and the presence of specific genes was detected and measured using qRT-PCR.

### Western blot analysis

Total protein in samples was extracted by RIPA lysis buffer (Keygen Biotech, Nanjing, China). The extracted proteins were first separated by 10% SDS-PAGE and transferred onto a PVDF membrane (Millipore) using standard procedures. PVDF membranes were blocked with 5% skimmed milk powder for 1 h and then incubated overnight at 4 °C with primary antibodies. The antibodies used in this study were CD63, Alix, HSP70, JAM3, and GAPDH (Abcam, Cambridge, UK). PVDF membranes were then probed with HRP-labeled secondary antibody (Santa Cruz, Dallas, TX, USA) for 1 h and signals were detected by chemiluminescence.

### Xenograft nude mouse model

Adult male BALB/C nude mice (6–8 weeks of age, *n* = 5/group) were purchased from the Laboratory Animal Center of Zhejiang University and were maintained under specific pathogen-free conditions with a 12-h light/dark cycle. All animal studies were approved by the Institutional Animal Care and Use Committee of the First Affiliated Hospital, College of Medicine at Zhejiang University and carried out in accordance with institutional and national guidelines. SMMC-7721 cells stably transfected with sh-NC or sh-circRNA, or treated with WT Mφ-Exo, RBPJ^+/+^ Mφ-Exo or RBPJ^+/+^ Mφ-Exo-sh-circRNA (5 μg/ml) were subcutaneously injected into the right upper back of the nude mice (1 × 10^6^ cells per mouse). When a tumor was palpable, tumor growth was measured every 4 days for 28 days with a caliper, and tumor volumes calculated by the formula: volume = (length × width^2^)/2. After 4 weeks, the mice were sacrificed and the tumors were dissected and weighed. Tumor tissues were collected for examination.

### Statistical analysis

Data are presented as the means ± standard deviation (SD). All experiments were performed at least in triplicate. The Student’s *t*-test and the one-way analysis of variance (ANOVA) were used to analyze the statistical significance as appropriate. Correlations were calculated using Pearson’s correlation analysis. *P*-values < 0.05 were considered statistically significant.

## Results

### Exosomes derived from macrophages overexpressing RBPJ inhibit proliferation and promote apoptosis in HCC cells

**Fig. 1:**
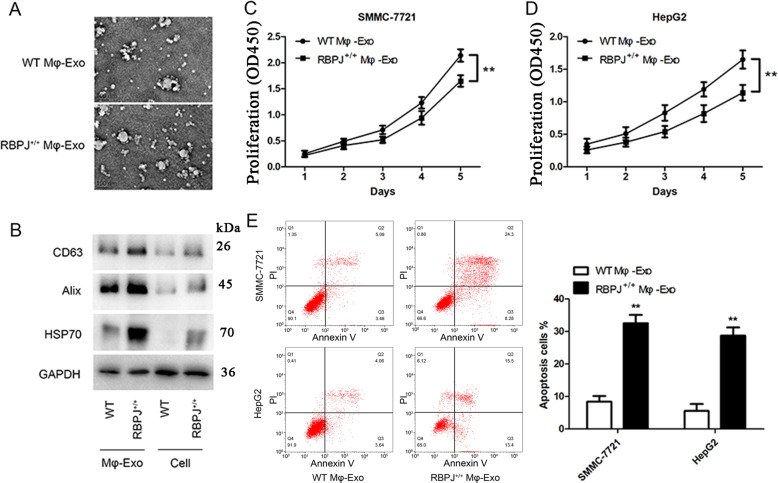
Characterization of exosomes and the co-culture of RBPJ-overexpressed macrophage-derived exosomes inhibited the proliferation and induced the apoptosis of hepatocellular carcinoma (HCC) cells. **A** Exosomes isolated from WT THP-1 derived macrophages (WT Mφ-Exo) and RBPJ-overexpressed macrophages (RBPJ^+/+^ Mφ-Exo) imaged by transmission electron microscopy (TEM) are approximately 100 nm in size. Scale bar = 100 nm. **B** Levels of exosome markers CD63, Alix, TSG101 and HSP70 in WT or RBPJ^+/+^ Mφ-Exo and WT or RBPJ^+/+^ Mφ (Cell) were determined by Western blotting. **C**, **D** Cell proliferation in HCC cell lines SMMC-7721 and HepG2 treated with exosomes derived from RBPJ-overexpressed macrophages was assessed by a CCK-8 assay. **E** Flow cytometry is performed to indicate cell apoptosis. The percentage of apoptosis was then measured. All experiments were performed three times. **P < 0.01, as indicated.

Exosomes from WT THP-1 derived macrophages (WT Mφ-Exo) and macrophages overexpressing RBPJ (RBPJ^+/+^ Mφ-Exo) were isolated by ultracentrifugation and characterized by TEM. In TEM images exosomes were 30–100 nm in diameter and had a round cup-like concave morphology (Fig. 1A). To further confirm the identity of the exosomes, the expression levels of exosome markers, CD63, Alix and Hsp70, were assessed. Western blot analysis showed that the isolated exosomes were enriched with CD63, Alix and HSP70 (Fig. 1B). These data indicate the successful isolation of exosomes from THP-1-like macrophages (WT Mφ-Exo) and RBPJ-overexpressed macrophages (RBPJ^+/+^ Mφ-Exo).

To further investigate the effects of these two groups of exosomes on the proliferation of HCC cells, we cocultured SMMC-7721 or HepG2 cells with exosomes for 5 days and measured cell proliferation through CCK-8 assays. As demonstrated in Fig. 1C, D, the presence of RBPJ^+/+^ Mφ-Exo significantly inhibits the proliferation of SMMC-7721 and HepG2 cells when compared to WT Mφ-Exo. In addition, flow cytometry indicates that RBPJ^+/+^ Mφ-Exo were able to induce cell apoptosis in SMMC-7721 and HepG2 cells (Fig. 1E). Overall, these results confirm that the overexpression of RBPJ in exosomes can inhibit proliferation and induce apoptosis in HCC cells.

### Expression profiles of circRNAs in exosomes derived from macrophages with RBPJ overexpressed

**Fig. 2:**
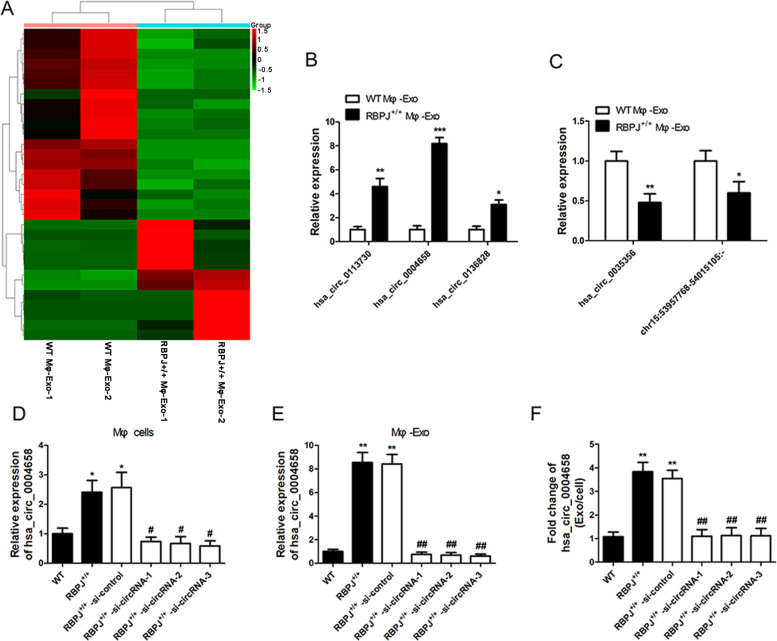
circRNA expression profiles in exosomes derived from RBPJ-overexpressed macrophages. **A** Cluster heatmap showing 31 aberrantly expressed circRNAs, including 12 upregulated and 19 downregulated circRNAs in exosomes derived from RBPJ-overexpressed macrophages and controls. The red color represents high expression, whereas the green color represents low expression. **B**, **C** The relative expression of three most upregulated circRNAs (hsa_circ_0113730, hsa_circ_0004658, and hsa_circ_0136828) and two most downregulated circRNAs (hsa_circ_0035356 and chr15:53957768–54015105) were validated by qRT-PCR (*n* = 5). *P < 0.05, **P < 0.01, ***P < 0.01, vs. the WT Mφ-Exo group. **D**, **E** The qRT-PCR assay indicated the difference in the hsa_circ_0004658 expression in WT, RBPJ-overexpressed Mφ cells with or without si-circRNA **D**, as well as in exosomes from those cells **E**. **F** The fold change of has_circ_0004658 expression between the exosomes and their corresponding producer cells. *, #*P* < 0.05, **, ##*P* < 0.01, as indicated. * vs. the WT group, # vs. the RBPJ^+/+^ group.

The circRNA profiles in exosomes derived from macrophages with RBPJ overexpressed and THP-1-like macrophages were evaluated using a circRNA microarray technique. Among the 4354 circRNAs that were detected, 31 circRNAs were differentially expressed (*P* < 0.05 and fold-change > 2.0) between exosomes derived from RBPJ-overexpressed macrophages and the controls (Fig. 2A). Of the 31 circRNAs, 12 were significantly upregulated and 19 were significantly downregulated. The three most upregulated circRNAs (hsa_circ_0113730, hsa_circ_0004658, and hsa_circ_0136828) and two most downregulated circRNAs (hsa_circ_0035356 and chr15:53957768-54015105) were selected and validated to be present in RBPJ^+/+^ Mφ-Exo and WT Mφ-Exo by qRT-PCR (Fig. 2B, C). Among the five circRNAs that had the greatest differential expression in RBPJ-overexpressed macrophages, hsa_circ_0004658 was selected as a candidate circRNA because it was upregulated with the highest significance. The expression of hsa_circ_0004658 is significantly higher in the RBPJ-overexpressed Mφ cells (2.2-fold) and exosome (8.2-fold) than in the WT Mφ cells (Fig. 2D, E). Compared with those in the producer cells, the levels of hsa_circ_0004658 are enriched by approximately 3.8-fold in the exosomes derived from RBPJ-overexpressed Mφ cells (Fig. 2F). Bioinformatics analysis found that hsa_circ_0004658 (chr18:2890558-2892484) was derived from elastin microfibril interface 2 (EMILIN2), which is located on chromosome 18p11.32.

### Mφ-Exo-hsa_circ_0004658 inhibits proliferation and migration and promotes apoptosis in HCC cells

**Fig. 3:**
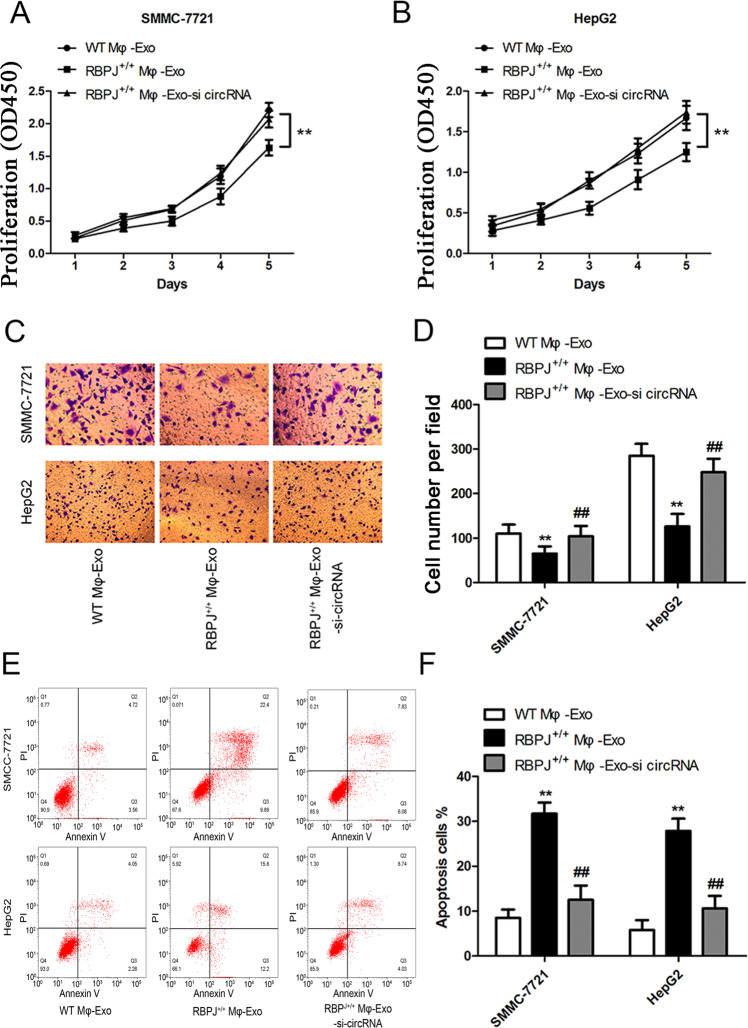
Mφ-Exo-hsa_circ_0004658 inhibits proliferation and migration and promotes apoptosis in hepatocellular carcinoma (HCC) cells. To remove hsa_circ_0004658 from exosomes, siRNA of hsa_circ_0004658 was transfected into THP-1 cells and Mφ-Exo were collected at 48 h post-transfection (RBPJ^+/+^ Mφ-Exo-si-circRNA). HCC cell lines SMMC-7721 and HepG2 were cocultured with WT Mφ-Exo, RBPJ^+/+^ Mφ-Exo or RBPJ^+/+^ Mφ-Exo-si-circRNA (5 μg/ml). **A**, **B** Cell proliferation in HCC cell lines SMMC-7721 and HepG2 was assessed by a CCK-8 assay. **C**, **D** Cell migration in HCC cell lines SMMC-7721 and HepG2 was assessed by Transwell assay. **E**, **F** Cell apoptosis in HCC cell lines SMMC-7721 and HepG2 was assessed by flow cytometry. All experiments were performed three times. **, ##*P* < 0.01, as indicated. * vs. WT Mφ-Exo, # vs. RBPJ^+/+^ Mφ-Exo.

The siRNA against hsa_circ_0004658 was constructed and transfected into Mφ cells and exosomes were collected at 48 h post-transfection. Next, the proliferation and migration of HCC cells were investigated by coculturing cells with exosomes derived from the macrophages (Fig. 3A–D). The inhibitory effects of RBPJ^+/+^ Mφ-Exo on the proliferation and migration of HCC cells (SMMC-7721 and HepG2) were eliminated when hsa_circ_0004658 was knocked down in exosomes (Fig. 3A–D). In addition, knockdown of hsa_circ_0004658 also significantly reduced the apoptosis induced by RBPJ^+/+^ Mφ-Exo in SMMC-7721 and HepG2 cells (Fig. 3E, F). This would be expected if there was an association between the expression of RBPJ and hsa_circ_0004658.

**Fig. 4:**
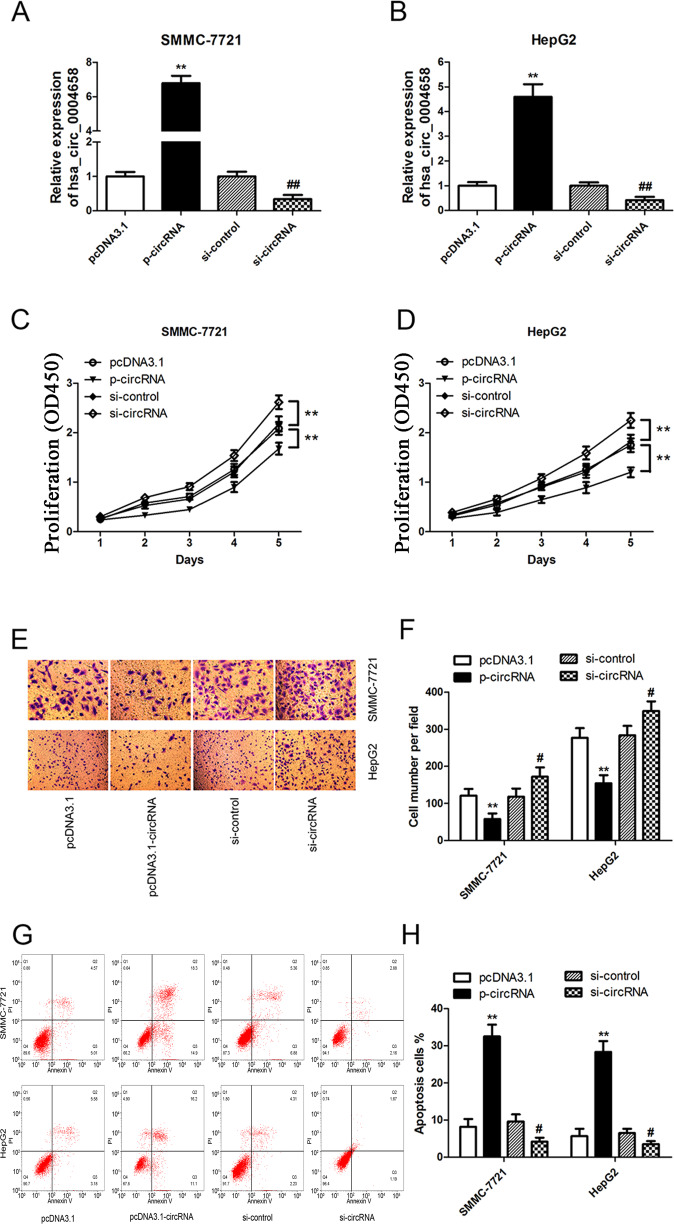
hsa_circ_0004658 inhibits proliferation and migration and promotes apoptosis in hepatocellular carcinoma (HCC) cells. **A**, **B** SMMC-7721 and HepG2 cells were transfected with hsa_circ_0004658 overexpression plasmids and hsa_circ_0004658 siRNA. The level of hsa_circ_0004658 was determined by RT-PCR in SMMC-7721 **A** and HepG2 **B** cells. **C**, **D** Cell proliferation in HCC cell lines SMMC-7721 and HepG2 was assessed by a CCK-8 assay. **E**, **F** Cell migration in HCC cell lines SMMC-7721 and HepG2 was assessed by Transwell assay. **G**, **H** Cell apoptosis in HCC cell lines SMMC-7721 and HepG2 was assessed by Flow cytometry. All experiments were performed three times. #*P* < 0.05, **, ##*P* < 0.01, as indicated. * vs. pcDNA3.1, # vs. si-control.

To further clarify the biological role of hsa_circ_0004658 in HCC cells, SMMC-7721 and HepG2 cells were transfected with either a hsa_circ_0004658 overexpression vector or hsa_circ_0004658 siRNA (Fig. 4A, B). The proliferation and migration of HCC cells were significantly inhibited by the overexpression of hsa_circ_0004658 but clearly enhanced when hsa_circ_0004658 expression was blocked (Fig. 4C–F). Furthermore, apoptosis in HCC cells was induced by the overexpression of hsa_circ_0004658 and was relatively decreased when hsa_circ_0004658 was downregulated (Fig. 4G, H). As with the overexpression of RBPJ, the overexpression of hsa_circ_0004658 promotes apoptosis and inhibits proliferation in HCC.

### Hsa_circ_0004658 acts as a sponge for miR-499b-5p

**Fig. 5:**
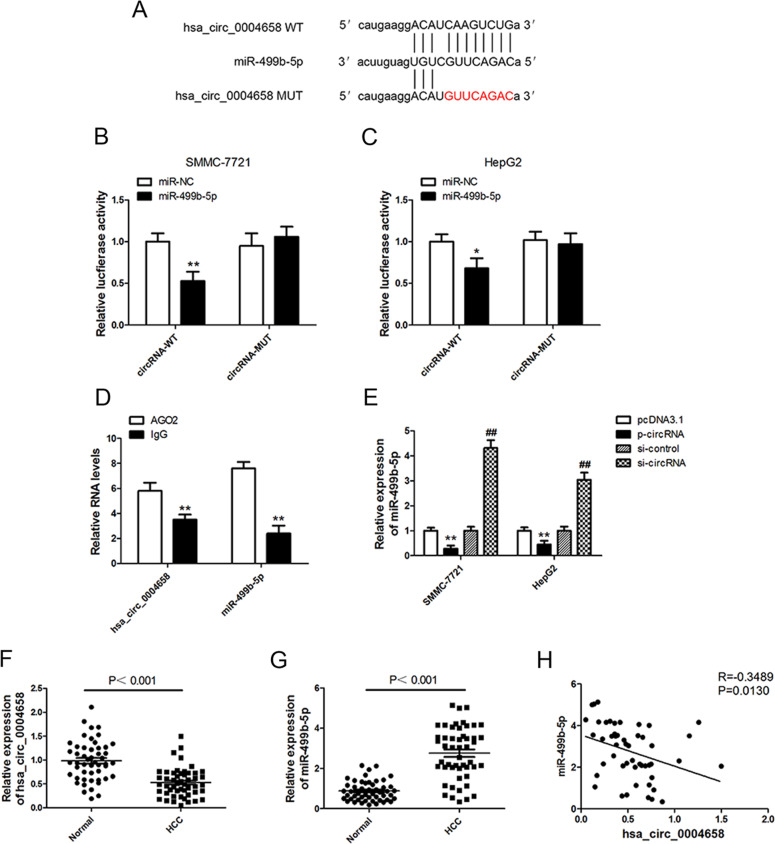
hsa_circ_0004658 acts as a sponge for miR-499b-5p. **A** Putative complementary sites within miR-499b-5p and hsa_circ_0004658 were predicted by bioinformatics analysis. **B**, **C** Dual-luciferase reporter assays demonstrate that miR-499b-5p is a direct target of hsa_circ_0004658 in SMMC-7721 **B** and HepG2 **C** cells (*P < 0.05, **p < 0.01). **D** The Ago2 RIP showed that Ago2 significantly enriched hsa_circ_0004658 and miR-499b-5p (**p < 0.01). **E** The level of miR-499b-5p was determined by RT-PCR in SMMC-7721 and HepG2 cells with control vector or circRNA overexpression plasmid, si-control or si-circRNA. **, ##*P* < 0.01, as indicated. * vs. pcDNA3.1, # vs. si-control. **F** The expression level of hsa_circ_0004658 in 50 hepatocellular carcinoma (HCC) tissues and matched para-carcinoma normal tissues was determined by qRT-PCR. **G** The expression level of miR-499b-5p in the above tissues was determined by qRT-PCR. **H** The expression levels of miR-499b-5p negatively correlated with hsa_circ_0004658 in HCC tissues.

To discover more about the specific regulation of hsa_circ_0004658 we searched two public databases (CIRCBASE and STARBASE, version 3.0) for interacting partners. Bioinformatic analysis predicted that hsa_circ_0004658 and miR-499b-5p possessed complementary binding sites (Fig. 5A). We used a dual-luciferase assay in HCC cells to confirm this interaction by mutating the predicted binding site in hsa_circ_0004658. Reduced luciferase activity was only observed in presence of wildtype hsa_circ_0004658 and miR-499b-5p in both SMMC-7721 and HepG2 cells (Fig. 5B, C). This was further validated using an Ago2 RIP assay. Ago2 significantly enriched RNA levels of both hsa_circ_0004658 and miR-499b-5p (Fig. 5D). Moreover, the levels of miR-499b-5p were significantly lower in cells overexpressing hsa_circ_0004658 and higher in cells with hsa_circ_0004658 silenced (Fig. 5E). This indicates that the expression of miR-499b-5p was strongly influenced by the level of hsa_circ_0004658. Levels of hsa_circ_0004658 and miR-499b-5p were also analyzed in HCC and matched para-carcinoma tissues. The results further substantiated that both hsa_circ_0004658 (Fig. 5F) and miR-499b-5p (Fig. 5G) had opposing regulatory roles in HCC. A negative correlation between miR-499b-5p and hsa_circ_0004658 was confirmed by regression analysis (Fig. 5H). This indicates that hsa_circ_0004658 may act as a sponge to competitively bind miR-499b-5p and prevent it from regulating other pathways.

### hsa_circ_0004658 inhibits the proliferation and induces apoptosis of HCC cells via the miRNA-499b-5p/JAM3 pathway

**Fig. 6:**
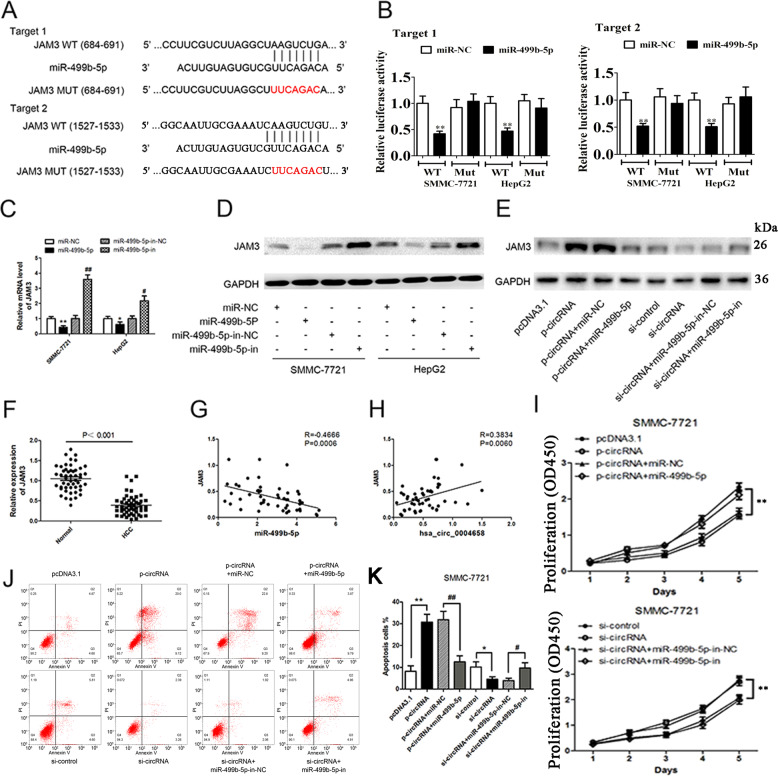
hsa_circ_0004658 inhibits proliferation and promotes apoptosis of hepatocellular carcinoma (HCC) cells by sponging miR-499b-5p and upregulating JAM3 expression. **A** Bioinformatics analysis revealed the predicted binding sites between JAM3 and miR-499b-5p. **B** Luciferase reporter assay demonstrated miR-499b-5p mimics significantly decreased the luciferase activity of JAM3-wt in SMMC-7721 and HepG2 cells. **C**, **D** The mRNA **C** and protein **D** level of JAM3 was detected through qRT-PCR and western blotting after treatment in SMMC-7721 and HepG2 cells. * vs. miR-NC, # vs. MiR-499b-5p-in-NC. **E** hsa_circ_0004658 overexpression upregulated JAM3 and siRNA downregulated JAM3, this effect can be reversed by co-transfection with miR-499b-5p mimics and miR-499b-5p inhibitors (miR-499b-5p-in) in SMMC-7721 cells respectively. **F** The expression level of JAM3 in 50 HCC tissues and matched para-carcinoma normal tissues was determined by qRT-PCR. **G** Expression levels of JAM3 negatively correlated with miR-499b-5p in HCC tissues. **H** Expression levels of JAM3 positively correlated with hsa_circ_0004658 in HCC tissues. **I** Cell proliferation in HCC cell lines SMMC-7721 was assessed by using a CCK-8 assay. **J**, **K** Cell apoptosis in HCC cell lines SMMC-7721 was assessed by flow cytometry. All experiments were performed three times and the representative data are shown with the corresponding *P*-values observed. *, #*P* < 0.05, **, ##*P* < 0.01, as indicated.

We next investigated the potential binding sites of miR-499b-5p. A miRNA target prediction and analysis using TargetScan (http://www.targetscan.org/vert_71/) identified that miRNA-499b-5p may interact with JAM3. JAM3 is a junctional adhesion molecule that has been implicated in several cancers. We mutated two potential miR-499b-5p target sites in JAM3 (Fig. 6A) and performed a luciferase reporter assay. The reporter assay demonstrated that the overexpression of miR-499b-5p in SMMC-7721 and HepG2 cells significantly decreased the luciferase activity of JAM3 at both target sites, and confirmed the predicted interaction (Fig. 6B). The levels of JAM3 mRNA and protein were reduced in SMMC-7721 and HepG2 cells overexpressing miR-499b-5p but were elevated in HCC cells not expressing miR-499b-5p (Fig. 6C, D). Overexpression of hsa_circ_0004658 upregulated JAM3 whereas cotransfection with miR-499b-5p downregulated JAM3 in SMMC-7721 cells (Fig. 6E). The opposite occurred when either hsa_circ_0004658 or miR-499b-5p was inhibited, JAM3 was down and upregulated, respectively. In HCC and matched para-carcinoma tissues, the expression of JAM3 was downregulated in HCC (Fig. 6F). In HCC tissue, JAM3 was negatively correlated with miR-499b-5p, whereas, it was positively correlated to hsa_circ_0004658 expression (Fig. 6G, H). Cell proliferation was increased and apoptosis was reduced in SMMC-7721 cells overexpressing miR-499b-5p and hsa_circ_0004658, and the opposite occurred when either hsa_circ_0004658 or miR-499b-5p were inhibited (Fig. 6I–K), leading to the assumption that hsa_circ_0004658 inhibits proliferation and promotes apoptosis in HCC cells by sponging miR-499b-5p and downregulating JAM3 expression.

### RBPJ^+/+^ Mφ-Exo inhibits tumor growth through the hsa_circ_0004658/miR-499b-5p/JAM3 pathway in vivo

**Fig. 7:**
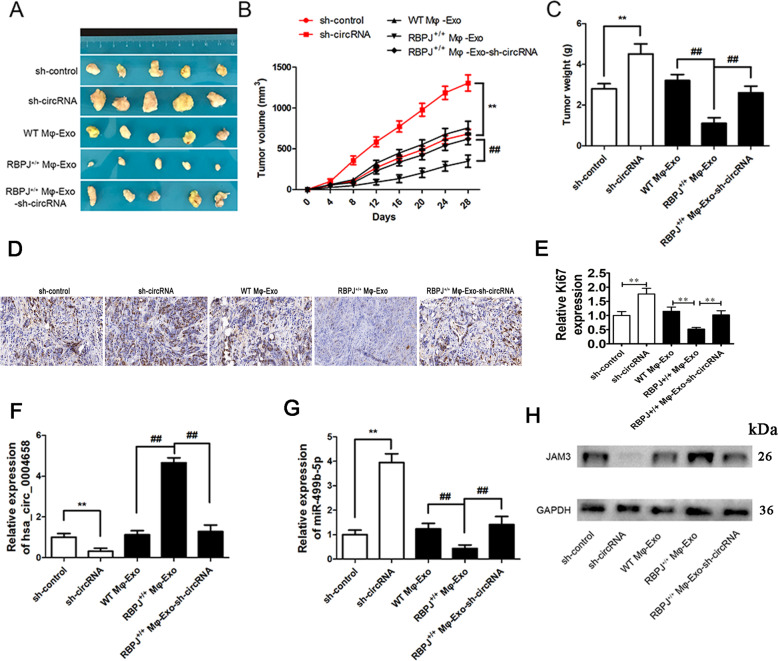
RBPJ+/+ Mφ-Exo inhibit tumor growth by hsa_circ_0004658/miR-499b-5p/JAM3 pathway in mouse xenograft tumor model. **A** Representative images of xenograft tumors (five mice per group) in nude mice. **B** Tumor volume is monitored every 4 days for 28 days. **C** The weights of xenograft tumors are summarized. **D**, **E** Immunohistochemical verification of the expression of Ki67 in tumors tissues. **, ## *P* < 0.01. **F**, **G** qRT-PCR verification of the expression of hsa_circ_0004658 **F** and miR-499b-5p **G** in tumors (*n* = 5). **, ##*P* < 0.01, * vs. sh-control, # vs. RBPJ^+/+^ Mφ-Exo. **H** The protein expression of JAM3 was detected by western blotting in tumors (*n* = 5).

To verify the effect of Mφ-Exo-hsa_circ_0004658 on the regulation of HCC growth in vivo, SMMC-7721 cells transfected with sh-circRNA or sh-control or cocultured with WT Mφ-Exo, RBPJ^+/+^ Mφ-Exo or RBPJ^+/+^ Mφ-Exo-sh-circRNA were subcutaneously injected into nude mice. The greatest difference in the tumor volume was between tumors of SMMC-7721 cells cocultured with RBPJ^+/+^ Mφ-Exo and those transfected with sh-circRNA (Fig. 7A–C), whereas sh-circRNA tumors were significantly larger. Immunohistochemical for Ki67 staining also show that Ki67 expression in sh-circRNA tumors were significantly higher (Fig. 7D–E). The relative expression of hsa_circ_0004658 was the highest in the RBPJ^+/+^ Mφ-Exo tumors and the lowest in sh-circRNA tumors (Fig. 7D) and in contrast, the relative expression of miR-499b-5p was the highest in sh-circRNA tumors and lowest in RBPJ^+/+^ Mφ-Exo tumors (Fig. 7E). Consequently, protein levels of JAM3 are highest in RBPJ^+/+^ Mφ-Exo tumors and lowest in sh-circRNA tumors (Fig. 7F). These results signify that RBPJ^+/+^ Mφ-Exo inhibits tumor growth through a hsa_circ_0004658/miR-499b-5p/JAM3 pathway in xenograft tumor models.

## Discussion

Macrophages are abundant in the HCC tumoral environment and are associated with chronic liver inflammation [33, 34]. Moreover, the macrophage environment is heterogenous with the progression of tumors dependent on alternatively polarized M2 macrophages and tumorigenic immune responses dependent on M1-polarized macrophages [8, 35]. Therefore, improving the understanding of macrophage regulation in the tumoral environment is important in developing effective therapies against HCC. Notch-RBPJ signaling is believed to regulate TLR-induced inflammatory macrophage polarization by the indirect regulation of M1-specific genes [29].

In this study, we examined whether overexpressing RBPJ in macrophages would influence HCC cells. We found that exosomes derived from macrophages overexpressing RBPJ could inhibit proliferation and promote apoptosis in HCC cells. We further explored the associated interactions by investigating circRNAs that may be differentially regulated when RBPJ is upregulated in macrophages-derived exosomes. Using a circRNA microarray technique we discovered that 31 of 4354 exosomal circRNAs were differentially regulated in THP-1-like macrophages when RBPJ was overexpressed, 12 were upregulated and 19 were downregulated. We selected the five with the highest significance (hsa_circ_0113730, hsa_circ_0004658, hsa_circ_0136828, hsa_circ_0035356, and chr15:53957768-54015105) for further analysis and of these circRNA, hsa_circ_0004658, which is associated with EMILIN2, seemed to give the most consistent results. EMILIN2 is an extracellular matrix protein that promotes angiogenesis by binding to the epidermal growth factor receptor and its ablation can reduce the growth of tumors in a mouse model [36]. EMLIN2 is also thought to suppress the proliferation and migration of breast cancer cells by regulating the Wnt signaling pathway [37]. RBPJ is also associated with the Wnt signaling pathway [38] and Notch1 is thought to be involved in the regulation of macrophages through the Wnt pathway in HCC [30]. In our study, exosomes from macrophage overexpressing hsa_circ_0004658 (Mφ-Exo-hsa_circ_0004658) were able to inhibit proliferation and migration and promote apoptosis in HCC cells. These associations required further investigation and therefore we searched for miRNAs that may interact with hsa_circ_0004658.

Two public databases (CIRCBASE and STARBASE) predicted that hsa_circ_0004658 may interact with miR-499b-5p and luciferase assays were used to validate the prediction. Our differential expression studies found that miR-499b-5p and hsa_circ_0004658 appeared to have opposing effects on HCC cells. The overexpression of hsa_circ_0004658 seemed to reduce levels of miR-499b-5p. A similar effect was observed in HCC and matched para-carcinoma tissues, with a negative correlation existing between miR-499b-5p and hsa_circ_0004658. We deduced that hsa_circ_0004658 may repress miR-499b-5p to prevent it from interacting in other pathways. TargetScan revealed that miR-499b-5p interacted with JAM3. JAM3 is a junctional adhesion molecule that is associated with several cancers and is thought to function as a tumor suppressor in colorectal cancer [39]. Additionally, JAM3 suppresses apoptosis and promotes migration in renal carcinoma [40]. In the present study, we found that miR-499b-5p suppresses JAM3 expression increasing proliferation and inhibiting apoptosis. The overexpression of hsa_circ_0004658 leads to the sponging of miR-499b-5p, this then upregulates JAM3 to inhibit proliferation and promote apoptosis in HCC cells. Our results agree with those suggesting that JAM3 acts as a tumor suppressor [39]. JAM3 was downregulated in primary colorectal cancer tissues and this was thought to be influenced by methylation [41, 42]. The methylation status of JAM3 has been proposed as a marker for cervical cancer [41]. It could be worth investigating the methylation status of JAM3 in HCC tissue and the influence miRNAs have on epigenetic modifications to examine how the mechanisms function in more detail. In our study the expression of JAM3 was negatively correlated with miR-499b-5p levels in HCC tissue. In the tumors overexpressing RBPJ^+/+^ Mφ-Exo tumors were significantly smaller than all other tumors, therefore the upregulation of hsa_circ_0004658, which leads to the downregulation of miR-499b-5p and the upregulation of JAM3, results in a tumor-suppressive phenotype in the mouse HCC model.

To conclude, in this study we found that hsa_circ_0004658 inhibits proliferation and induces apoptosis of HCC cells via the miRNA-499b-5p/JAM3 pathway. Moreover, RBPJ^+/+^ Mφ-Exo inhibits tumor growth by upregulating the hsa_circ_0004658/miR-499b-5p/JAM3 pathway in vivo. Our results indicate that RBPJ^+/+^ Mφ-Exo may have potential in the regulation of tumor proliferation and hsa_circ_0004658 could be a diagnostic biomarker and potential target for HCC therapy.

## Supplementary information

aj-checklist

cddis-author-contribution-form

## Data Availability

The data that support the findings of this study are available from the corresponding author upon reasonable request.
