# Supplementary material for: Exosomal hsa_circ_0004658 derived from RBPJ overexpressed-macrophages inhibits hepatocellular carcinoma progression via miR-499b-5p/JAM3
Source: Cell Death Dis. 2022 Jan 10;13(1):32. doi: 10.1038/s41419-021-04345-9 (PMC8748962; doi:10.1038/s41419-021-04345-9)
Supplement: Supplementary file 2 — cddis-author-contribution-form [file 41419_2021_4345_MOESM2_ESM.pdf]

DECLARATION OF CONTRIBUTIONS TO ARTICLE

ADMC

Manuscript Number:

Journal Name: *Cell Death & Disease* (the 'Journal')

Proposed Title of the Contribution:

Exosomal hsa_circ_0004658 derived from RBPJ overexpressed-macrophages inhibits hepatocellular carcinoma progression via miR-499b-5p/JAM3 (the 'Contribution)

Author(s):

Lei Zhang, Jing Zhang, Pengfei Li, Ting Li, Zhiqin Zhou, Huiling Wu (the 'Authors')

For all *CDDis* articles, each person named as an author in the published version must be able to show he or she has contributed substantially to the article.

Authorship credit should be based on 1) substantial contributions to conception and design, acquisition of data, or analysis and interpretation of data; 2) drafting the article or revising it critically for important intellectual content; and 3) final approval of the version to be published. Authors should meet conditions 1, 2 and 3.

Any person who cannot be shown to have made a substantial contribution to the article cannot be listed as an author in the final version. The name of any person who is deemed to have made a minor contribution can, however, appear in the Acknowledgments section of the article.

Please complete the table below to indicate the contributions of all named authors to the manuscript.

Author Full Name:	Specification of Contribution to the Manuscript:
Lei Zhang	carried out the studies, participated in collecting data, and drafted the manuscript
Huiling Wu	carried out the studies, participated in collecting data, and drafted the manuscript
Jing Zhang	carried out the studies, participated in collecting data, and drafted the manuscript
Pengfei Li	performed the statistical analysis and participated in its design
Zhiqin Zhou	performed the statistical analysis and participated in its design
Ting Li	performed the statistical analysis and participated in its design

Please complete the table below to indicate the contributions of all named authors to the figures.

Figure 1:

Lei Zhang, Huiling Wu and Jing Zhang

Figure 2:

Lei Zhang, Huiling Wu and Jing Zhang

Figure 3:

Lei Zhang, Huiling Wu and Jing Zhang

Figure 4:

Pengfei Li, Zhiqin Zhou and Ting Li

Figure 5:

Pengfei Li, Zhiqin Zhou and Ting Li

Figure 6:

Pengfei Li, Zhiqin Zhou and Ting Li

Signed for and on behalf of the Author(s):	Print Name:	Date:
Huiling Wu	Huiling Wu	2021.05.13
